# Evaluation of two SpO_2_ alarm strategies during automated FiO_2_ control in the NICU: a randomized crossover study

**DOI:** 10.1186/s12887-019-1496-5

**Published:** 2019-05-06

**Authors:** Malgorzata Warakomska, Thomas E Bachman, Maria Wilinska

**Affiliations:** 1 Department of Neonatology, Independent Public Clinical Hospital of Prof W, Orlowski 231 Czerniakowska str, 00-416 Warsaw, Poland; 2 Department Biomedical Technology, Faculty of Biomedical Engineering, Czech Technical University in Prague, Sitna 3105, 272 01 Kladno, Czech Republic; 3 Department of Neonatology, Centre of Medical Postgraduate Education, 231 Czerniakowska str, 00-416 Warsaw, Poland

**Keywords:** Oxygen saturation, Alarm fatigue, Automated oxygen control

## Abstract

**Background:**

Changes in oxygen saturation (SpO_2_) exposure have been shown to have a marked impact on neonatal outcomes and therefore careful titration of inspired oxygen is essential. In routine use, however, the frequency of SpO_2_ alarms not requiring intervention results in alarm fatigue and its corresponding risk. SpO_2_ control systems that automate oxygen adjustments (Auto-FiO_2_) have been shown to be safe and effective. We speculated that when using Auto-FiO_2_, alarm settings could be refined to reduce unnecessary alarms, without compromising safety.

**Methods:**

An unblinded randomized crossover study was conducted in a single NICU among infants routinely managed with Auto-FiO_2_. During the first 6 days of respiratory support a tight and a loose alarm strategy were switched each 24 h. A balanced block randomization was used. The tight strategy set the alarms at the prescribed SpO_2_ target range, with a 30-s delay. The loose strategy set the alarms 2 wider, with a 90-s delay. The effectiveness outcome was the frequency of SpO_2_ alarms, and the safety outcomes were time at SpO_2_ extremes (< 80, > 98%). We hypothesized that the loose strategy would result in a marked decrease in the frequency of SpO_2_ alarms, and no increases at SpO_2_ extremes with 20 subjects. Within subject differences between alarm strategies for the primary outcomes were evaluated with Wilcoxon signed-rank test.

**Results:**

During a 13-month period 26 neonates were randomized. The analysis included 21 subjects with 49 days of both tight and loose intervention. The loose alarm strategy resulted in a reduction in the median rate of SpO_2_ alarms from 5.2 to 1.6 per hour (*p* <  0.001, 95%-CI difference 1.6–3.7). The incidence of hypoxemia and hyperoxemia were very low (less than 0.1%-time) with no difference associated with the alarm strategy (95%-CI difference less than 0.0–0.2%).

**Conclusions:**

In this group of infants we found a marked advantage of the looser alarm strategy. We conclude that the paradigms of alarm strategies used for manual titration of oxygen need to be reconsidered when using Auto-FiO_2_. We speculate that with optimal settings false positive SpO_2_ alarms can be minimized, with better vigilance of clinically relevant alarms.

**Trial registration:**

Retrospectively registered 15 May 2018 at ISRCTN (49239883).

**Electronic supplementary material:**

The online version of this article (10.1186/s12887-019-1496-5) contains supplementary material, which is available to authorized users.

## Background

Pulse oximetry (SpO_2_) is the standard of care for monitoring oxygenation in the NICU. [1, 2] Changes in SpO_2_ exposure, particularly extremes associated with hypoxemia and hyperoxemia are associated with marked changes in morbidity and mortality. [3] Notwithstanding its utility, nurses find managing SpO_2_ levels challenging. [4–11] Frequent false positive SpO_2_ swings are associated with motion artifact and erratic poor perfusion. In addition to these artifacts, excursions outside the desired SpO_2_ target range are often transient and do not require intervention. For these reasons, in the NICU, SpO_2_ alarms are the most prevalent and also the most often ignored by nurses [4]. Alarm fatigue is defined as becoming desensitized to alarms as a result of frequent non-actionable alarms. It is considered a major hazard in the ICU. [7, 8] While selection of proper SpO_2_ alarm settings has been proposed as a mitigating solution [8–10] to excess alarms, this is a trade-off. Creation of alarm fatigue with its associated loss of vigilance must be balanced with the risk of missing or delaying response to clinically relevant events. There is some evidence that setting alarms tightly can result in better SpO_2_ control. [2] This is perhaps true in clinical studies, but others contend looser settings are more appropriated for routine care. [1] Finally situational improvisation by nurses, both from unit alarm guidelines for alarm settings and from desirable alarm response, is common. [6, 11]

Automated FiO_2_ control systems (Auto-FiO_2_) have become available and have been shown to be safe and effective. [12] Importantly with the advent of Auto-FiO_2_ a different paradigm is relevant when considering SpO_2_ alarm strategies. During periods of manual titration of FiO_2_, the alarms serve to alert the nurse that a change in FiO_2_ should be considered. During Auto-FiO_2_, the system is making reasoned adjustments to the FiO_2_ continuously. Alarms serve rather to alert the nurse that despite these FiO_2_ adjustments, SpO_2_ readings are still compromised and therefore other interventions should be considered. Interventions might include moving the SpO_2_ sensor, arousing the infant, or perhaps adjusting the baseline FiO_2_. When using Auto-FiO_2_ the need to adjust the FiO_2_ is infrequent, generally only a few times per day. With such infrequent need to adjust the FiO_2_, another potential hazard is created, over reliance on automation.

We previously reported on the first year’s experience with routine use of Auto-FiO_2_ at 5 centers in Poland. [13] One finding was that looser alarm settings reduced the perception of excessive alarms. Nevertheless in considering this finding we realized that the opportunity of reducing alarms not needing intervention must also be balanced with the risk of over reliance on automation and missing relevant events.

The aim of this prospective controlled study was to determine whether a loose alarm strategy could significantly reduce SpO_2_ alarm frequency without increasing over reliance on automation resulting in an increased exposure to SpO_2_ extremes.

## Methods

This was a single center study, conducted at the Independent Public Clinical Hospital of Prof W. Orlowski, in Warsaw Poland. The NICU is a tertiary care unit, with 8 beds, and 250 annual admissions, of which 96% are inborn. After reviews of the protocol, the parent information sheet and the Informed Consent document, the study was approved by the institution’s Research Bioethics Committee.

At the time of the study, and for several prior years, the unit used only one type of mechanical ventilator. (AVEA, Vyaire, Yorba Linda, CA, USA). All ventilators included the Auto-FiO_2_ option (CLiO2). The standard practice was to routinely use the Auto-FiO_2_ system when infants were initially intubated, and required supplemental oxygen. The system is also capable of noninvasive support and was used sometimes when infants were transitioning from intubation, and also later in their course of treatment, in the case of exacerbation and prevalent desaturations.

This was a crossover study of two alarm strategies, tight and loose. It started on the first day of life or on initiation of support with the Auto-FiO_2_ system and ended with a transition from respiratory support or after 6 days, whichever occurred first. Assignment to the alarm strategy for the first day was randomized, and then switched every 24 h. Infants were eligible for the study dependent on an anticipated duration of at least 2 days of respiratory support with the Auto-FiO_2_ system, written informed consent, and the availability of the research team. The initial alarm strategy was assigned based on a balanced block (4) table. There was no blinding of the prospective or actual intervention.

The SpO_2_ target range, set on the Auto-FiO_2_ system, was selected by the attending physician. The prevailing unit preference was a setting of 88–95% SpO_2_. The initial and daily changes to alarm settings were implemented by the research team. The tight strategy set the SpO_2_ alarms to trigger just outside the target range, nominally at 87 and 96% SpO_2_. The loose strategy set the thresholds 2 wider, nominally 85 and 98% SpO_2_. The SpO_2_ alarm delays were also different, 30 s and 90 s respectively.

The outcomes were selected prospectively. The primary effectiveness outcome was the relative frequency of audible SpO_2_ alarms. The primary safety outcomes were the prevalence (percent time) at extreme SpO_2_ levels. We defined these as hyperoxemia (SpO_2_ > 98% with FiO_2_ > 21%) and hypoxemia (SpO_2_ < 80%). Several secondary descriptive outcomes were also specified. Prolonged episodes of hypoxemia and hyperoxemia were defined as longer than 1 min and 3 min. In addition a liberal definition of normal SpO_2_ (86–96% SpO_2_) was retrospectively defined to be more inclusive of the variation in the actual set SpO_2_ control ranges. It was reported both as normoxemia which included time when SpO_2_ > 96% with a FiO_2_ of 0.21, and also only during periods of supplemental oxygenation. The median SpO_2_ and FiO_2_ were calculated for each hour, and reported as the mean of the median levels.

Data from the ventilator were collected on a data logger every 5 s. It was summarized with purpose build software. We have used these tools in previous studies. [14, 15]

We determined that we would be able to detect a drop in the alarm frequency of 50% associated with the loose strategy, assuming a within subject standard deviation of 75%, with 20 subjects (power > 0.80, *p* <  0.05).

Summary data were analyzed using XLSTAT v19.02 (Addinsoft, Paris, France). The data for the periods of tight and periods of loose alarm strategy were pooled for each subject. These data are presented as mean (STD) or median (IQR). Correspondingly within subject differences between alarm strategies were evaluated with paired t or Wilcoxon signed-rank test, as appropriate. A *p* < 0.05 was considered to be statistically significant. Effect sizes of the primary outcomes were also described with 95% confidence intervals of the median difference.

## Results

In a 13-month period starting in June 2016, thirty-three infants were treated with the Auto-FiO_2_ system. Of these 4 were not considered because of the absence of the research team, and 3 were excluded because their anticipated need for respiratory support was very short. The remaining 26 subjects all consented to participate and were enrolled in the study. Five of the 26 did not complete two days of intervention and were excluded from this analysis. Recruitment stopped when 20 subjects had completed the study.

The demographics of the remaining 21 subjects are summarized in Table 1. As shown, the subjects were mostly preterm infants. Their birth weights ranged between 0.60 and 3.3 kg. The study interventions began in the first or second day of life in all but 3 subjects. Those three subjects were 3, 3, and 26 days old at enrollment. Surfactant was administered in nearly all (15/17) of the infants less than 32 weeks gestational age. Two received a second dose. Each nurse was usually responsible for 2 infants such as those in the study, although staffing was not recorded. Sedation and analgesia are not used during respiratory support.

**Table 1:** Subject Demographics and Physiological Baseline

Parameter	
Number of subjects	21
Birth Weight (grams)	930 (800–1955)
Gestational Age (weeks)	28 (25–31)
Gender (female/male)	4/17
Any NCPAP prior to enrollment (%)	13 [62%]
Maximum FiO_2_ prior to enrollment (%)	50 (43–68)
FiO_2_ at enrollment (%)	33 (26–51)
Age greater than 2 days	3 (14%)

Median (IQR), frequency (%)

Details of respiratory support during intervention and the reason for exit are shown in Table 2. Most (17/21) were intubated at the start of the study and remained intubated until exit (14/17). None were moved from noninvasive to intubation during the study period. Many of the subjects (8/21) were exited before the 6-day limit. In addition 7 days of intervention were excluded for protocol violations (alarm settings were inconsistent with either alarm strategy). Thus 98 days of intervention were evaluated, 49 during the loose alarm strategy and 49 during the tight alarm strategy. The 8 subjects who exited prior to 6 days no longer needed respiratory support on the ventilator with Auto-FiO_2_. Most of these required a lower level of support. However, 2 were transferred to HFOV, one as a result of a pneumothorax and the other hypercapnia. There were no adverse events noted related to the protocol or Auto-FiO_2_ system. The only mode of noninvasive support was nasal CPAP. For intubated infants time-cycled, pressure-limited support was the predominant mode (65% A/C, 15% SIMV).

**Table 2:** Entrance and Exit

Parameter	
First day assignment (Tight/Loose)	12/9
Intubated at enrollment (%)	17 (81%)
Transitioned to NCPAP during study (%)	3 (14%)
Ventilation (noninvasive/invasive %)	23%/77%
Reason for Exit	
Completed 6 days	13 (62%)
Transferred to HFOV	2 (10%)
Transitioned from respiratory support	6 (29%)
Data not available	
Exit before 6 days (days)	21
Miss-set SpO_2_ alarms (days)	7
Total days of data analyzed (days)	98
days of data analyzed (Tight/Loose)	49/49

frequency (%)

**Fig.1:**
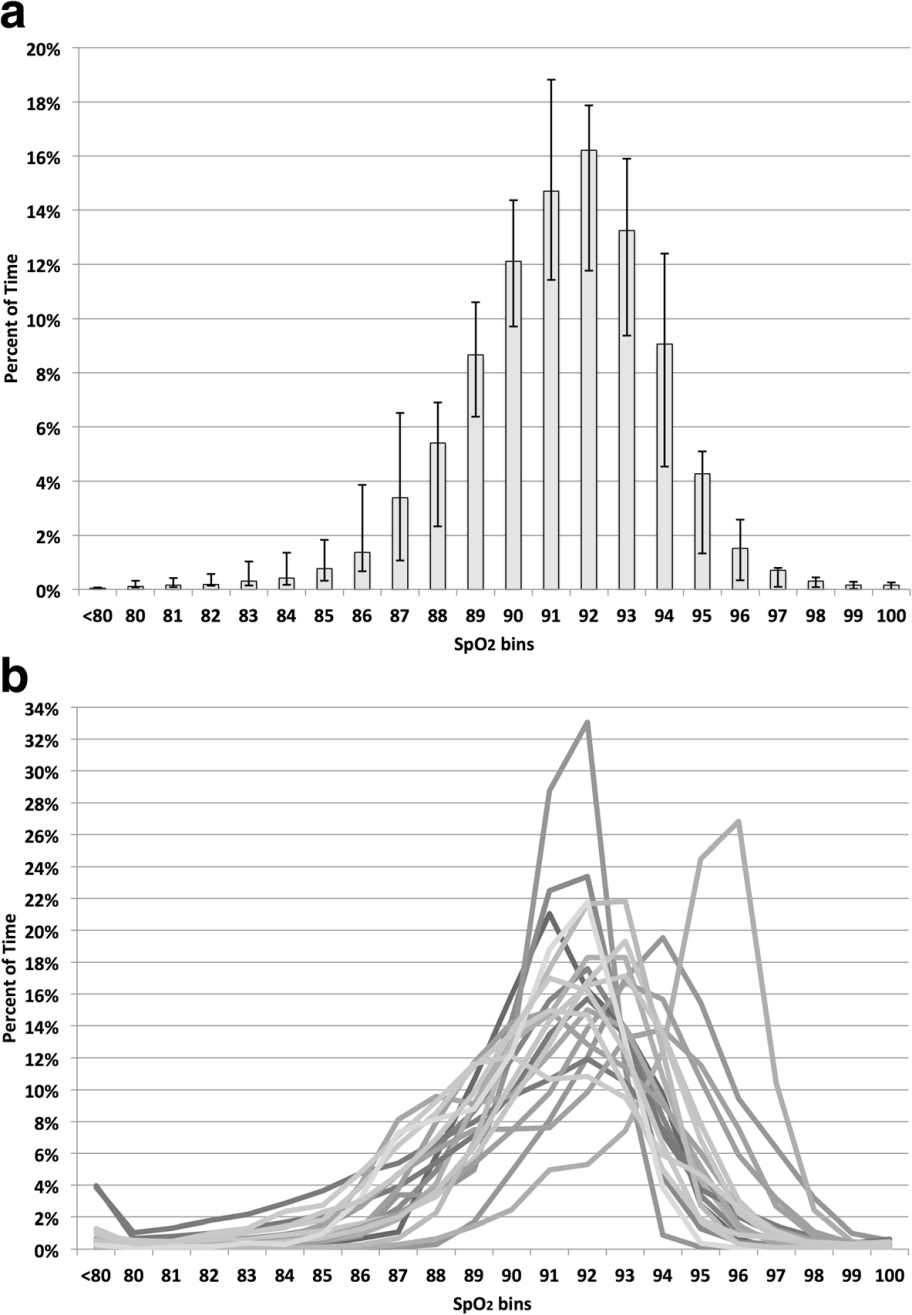
**a** SpO_2_ Histogram when FiO_2_ > 0.21. Bars are the median percent time, and whiskers their IQR **b** SpO_2_ Histogram of Each Subject when FiO_2_ > 0.21. The subject whose distribution is skewed to the right had an upper SpO_2_ control limit setting of 97%

Histograms of the SpO_2_ exposure during the study are shown in Fig.1a and b. Figure 1a shows the histogram of the median of the 21 subjects, including the IQR at each SpO_2_ level. The variability among subjects is further depicted in Fig. 1b; a SpO_2_ histogram for each of the 21 subjects. Among the 98 days of intervention the median SpO_2_ ranged between 89 and 96% and the FiO_2_ ranged between 0.21 and 0.67. The median and (IRQ) of the FiO_2_ at the initiation of the study was 0.33 (0.26–0.51). In the first day the changes in FiO_2_ ranged between a drop of 0.42 and an increase of 0.06. Across these changes in oxygenation requirements, Auto-FiO_2_ was quite effective. Manual FiO_2_ adjustments were infrequent, ranging between 0 and 14 per day. Further the percent time with SpO2 between 86 and 96% during supplement oxygen in ranged between 60 and 98%.

**Fig. 2:**
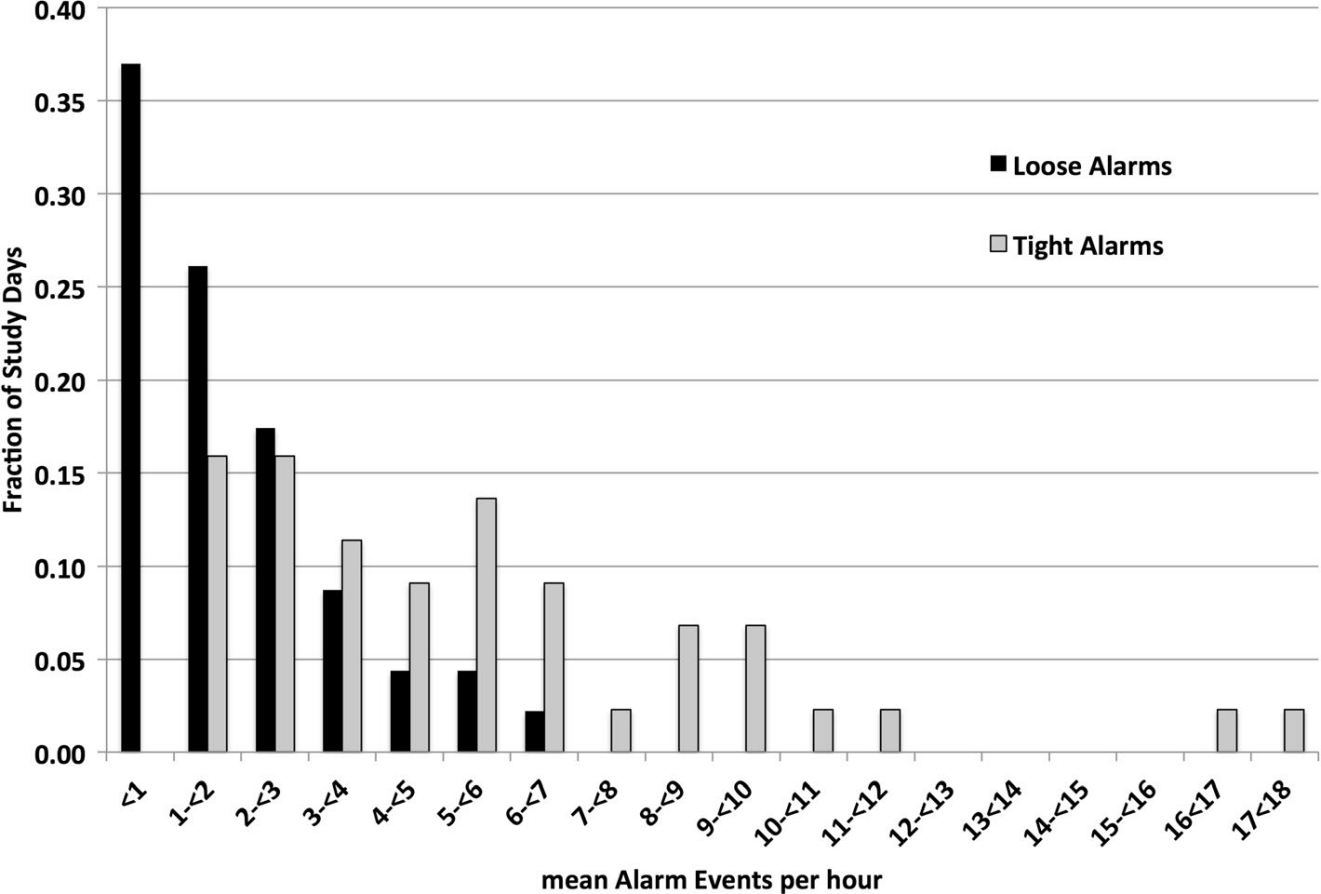
Histogram of SpO_2_ Alarm Events Reflects the hourly rate of events for each study day

The average Auto-FiO_2_ settings, inspired oxygen and oxygenation saturation for the 21 subjects are shown in Table 3, tabulated by the alarm strategy. There was no difference in the set auto control target range, nominally 88–95% SpO_2_. The differences in the alarm settings were as planned. During the loose alarm strategy the High and Low SpO_2_ alarms were each set about 2 wider than during the tight alarm strategy and the alarm delay was three times longer; 90 s as compared to 30 s. There were no differences in the median FiO_2_, time with normal oxygen saturation or manual adjustments of FiO_2_ associated with the two alarm strategies. The median SpO_2_ was slightly higher during the loose strategy, but both were near the mid point of the set control range.

**Table 3:** Course of Study Intervention

	Loose Alarm Strategy	Tight Alarm Strategy	P
Number of subjects	21	21	–
Automated FiO_2_ Settings			
High Target-Range (SpO_2_%)	95.1 (0.8)	95.0 (0.7)	ns
Low Target-Range (SpO_2_%)	88.4 (1.0)	88.5 (1.0)	ns
High SpO_2_ Alarm (SpO_2_%)	98.0 (0.9)	96.3 (0.8)	< 0.001
Low SpO_2_ Alarm (SpO_2_%)	85.6 (1.1)	87.4 (1.0)	< 0.001
SpO_2_ alarm delay (sec)	90.0 (2.9)	30.4 (1.7)	< 0.001
Median FiO_2_ (%)	28.1 (6.2)	30.7 (8.6)	ns
Median SpO_2_ (%)	92.5 (1.1)	92.1 (1.0)	0.039
SpO_2_ 86–96%* (%time)	89.7(6.8)	91.5 (8.9)	ns
Normoxemia** (%time)	95.2 (3.9)	94.9 (4.6)	ns
Manual FiO_2_ adjustments (/day)	2 (1–5)	2 (2–3)	ns

* during periods when FiO_2_ > 0.21, **Normoxemia is defined as SpO_2_ between 86 and 96% or > 96% if FiO_2_ = 0.21. P for paired comparison of the mean (SD) or median (25th–75th percentile) with paired t test or Wilcoxon signed-rank test

The study outcomes are shown in Table 4. The loose alarm strategy resulted in a 69% reduction in the frequency of SpO_2_ alarms (from median per hour of 5.2 to 1.6, *p* < 0.001, 95% CI difference 1.6–3.7). Reinforcing this difference, the alarm frequency range and frequency in individual days are shown in the descriptive histogram in Fig. 2. There are two alarm related exploratory variables in the table. First, the loose strategy also resulted in less total time with any alarm active (41% reduction *p* < 0.034). Second, even with these reductions, SpO_2_ alarms accounted for about half of all the alarms during the loose strategy. The difference in percent time at SpO_2_ extremes was not different, and the 95% confidence intervals of the differences were small (hypoxemia 0–0.2% and hyperoxemia − 0.1-0.1%). In addition there were no differences in the frequency of prolonged episodes of hypoxemia and hyperoxemia. Episodes of hyperoxemia of 3 min or longer were rare. During all 98 days there were only 14. There was a trend of more frequent episodes during use of the loose alarm strategy (11/14).

**Table 4:** Outcomes of Study Interventions

	Loose Alarm Strategy	Tight Alarm Strategy	P
Number of subjects	21	21	–
SpO_2_ Alarms (#/hour)	1.6 (0.8–2.6)	5.2 (3.0–6.6)	< 0.001
SpO_2_/all Alarms (%)	47 (30–75)	75 (64–91)	< 0.001
Audible alarm (% time)	6.9 (2.9–14.3)	11.7 (10.5–16.4)	0.034
Hypoxemia [SpO_2_ < 80%]			
Total (% time)	0.2 (0.1–0.4)	0.3 (0.1–0.8)	ns
Episodes> 1 min (#/24-h)	1.2 (0–2.2)	1.1 (0.3–2.2)	ns
Episodes> 3 min (#/24-h)	0 (0–0.4)	0 (0–0.5)	ns
Hyperoxemia [SpO_2_ > 98%]			
Total (% time)	0.2 (0–0.2)	0.2 (0.1–0.5)	ns
Episodes > 1 min (#/24-h)	0.7 (0–1.7)	0.5 (0–1.7)	ns
Episodes > 3 min (#/24-h)	0 (0–0)	0 (0–0)	ns

SpO_2_ > 98% excludes time when FiO_2_ = 0.21. Median (25th–75th percentile). P for paired comparison with Wilcoxon signed-rank test

## Discussion

SpO_2_ alarm fatigue is a major issue in the NICU. We evaluated the impact of setting SpO_2_ alarms looser during automated FiO_2_-SpO_2_ control. We found these looser settings dramatically reduced the frequency and duration of SpO_2_ alarms without compromising safety. To our knowledge this is the first study evaluating the impact of specific SpO_2_ alarm levels during automated FiO_2_-SpO_2_ control. A recent report on the results of a quality improvement effort reported similar reductions in non-actionable alarms during manual control associated with refinements to the target range, SpO_2_ alarm levels and alarm delays. [9]

An idealistic goal would be to set the breadth and time delay of alarms such that false alarms (i.e., those not needing intervention) were eliminated, without missing relevant events. While our loose alarm strategy was a marked improvement, its definition was arbitrary. It was based only on subjective experiences [13] and also impacted by the clinician selected SpO_2_ target range. In considering the optimum alarm strategy it is likely that the high level, and low level and time delay should each be considered separately. That is, each independent of the desired SpO_2_ target range, but rather associated with risks of oxygenation extremes. In our study the attending physician selected the set target range. It varied but was nominally 88–95%. One study suggests that a narrower set target range when using Auto-FiO2 is beneficial. [16] In that study comparing set target ranges during the use of the same Auto-FiO_2_ system that we used, van den Heuvel et al. found that 88–92% was preferred to either 86–94% or 89–91%, assuming a goal of reducing exposure to SpO_2_ extremes. Two studies have also shown that a modest shift in the median of the set target range during Auto-FiO_2_ has a marked effect on the SpO_2_ exposure. [14, 15]

A recent study of the SpO_2_-PaO_2_ relationship provides some perspective for high and low SpO_2_ alarm levels. [17] The likelihood of hypoxemia increases as SpO_2_ drops below 90% and is marked below 85%. The likelihood of hyperoxemia increases as SpO_2_ is above 95%. However it is different for preterm and near term infants. In preterm infants it is not marked until the SpO_2_ is above 98%. In contrast for near term infants it is marked above 96%. So these potential alarm levels (85 & 98% for preterms and 85 & 96% for near terms) represent the thresholds for a marked likelihood of oxygenation extremes that should be avoided. Tighter SpO_2_ alarm settings of the higher or lower threshold would provide a margin of error.

Finally the alarm delay needs to be considered. Poets et al., evaluated the relationship between hypoxemia (SpO_2_ < 80%) and long term outcomes. [18] They found that episodes longer than 1 min were the main cause of increased time in hypoxemia, which was also correlated with poor outcomes. These prolonged episodes, that impacted outcome, represented only 14% of all the episodes < 80% SpO_2_. We are unaware of any such careful analysis of the clinical impact of hyperoxemic episodes on outcome. Nevertheless it is clear that increased time at very high levels of SpO_2_ impact outcome. [3] In our study using Auto-FiO_2_ there were only a few episodes longer than 1 min per day. Another study of this Auto-FiO_2_ system found the number of these episodes seemed to be associated with the actual set control target range. [16] With all this in mind we speculate that there would be little utility in increasing the alarm delay beyond 90 s, and that reducing it to 60 s could be appropriate especially for the widest high and low SpO_2_ alarm levels. It should be noted that while high and low SpO_2_ alarms predominate in routine manual care, the oximeter also includes other alarms. These warn of poor signal quality and drop-out and can be prevalent. These conditions, when persistent, are certainly relevant to the need for clinical assessment. Our study was not designed to analyze the direct impact of alarm delay on signal quality alarms. Since the 90-s delay in the loose alarm strategy should have eliminated all but a few high and low SpO_2_ alarms each day, we speculate that the residue of a couple per hour are persistent signal quality alarms.

Nearly all of the studies on the effectiveness of Auto-FiO_2_ have been short-term crossover studies [19]. Studies during routine use, like ours, are limited. Our previous report on the general use of Auto-FiO_2_ with 121 infants in 5 Polish centers, described indications for use, typical settings and general outcomes and impressions, but not the quantitative effectiveness of SpO_2_ control. [13] Van Zanten et al. reported on their transition to routine use of Auto-FiO_2_ in a before-after study. [19] Their Auto-FiO_2_ arm included 21 preterm infants over a 5-month period. They were treated for a median of 11 days, predominately with noninvasive respiratory support often following an initial phase of invasive support. Compared to the crossover studies they reported lower levels of hypoxemia (median 0.9% time SpO_2_ < 80%) and hyperoxemia (median 2.1% time SpO_2_ > 98%). In our study we experienced even lower levels at these SpO_2_ extremes. We speculate that the difference is a reflection of the treatment populations. We studied mostly infants in the first week of life who were also more likely to be intubated. For these reasons they were probably more stable than those studied over their full course of respiratory support reported by van Zanten.

Our study has some limitations to consider with regard to generalizability. First the criteria defining both of the alarm strategies were selected based on our general experience and not based on a priori knowledge about the SpO_2_ exposure. Had the SpO_2_ target range been controlled, and the SpO_2_ alarm limits set independently, the results might have been more precise. However as suggested above, this would have resulted in reduced incidence of SpO_2_ extremes, which were already sparse. Second we studied infants according to our standard practice of use of the system, that is, mostly when intubated in the first days of life. This resulted in a population that was relatively stable, compared to infants later in life, or on noninvasive support. The infants we studied experienced about 5 desaturations per hour that triggered an alarm during the tight SpO_2_ alarm strategy. It is not certain whether the 69% reduction in SpO_2_ alarms might be anticipated in less stable infants. Although this seems to be a reasonable assumption, it should be prospectively studied. Third we averaged the response to the two strategies for each of 21 subjects, rather than treating each of the 98 days of use as independent parameters. This seems to us as the most conservative approach and provides statistical validity of a within subject paired evaluation. However the latter could have yielded different results, as more than a quarter of the subjects were weaned from the system in less than 6 days. Likewise the 7 days excluded for protocol violations is also of concern. Fourth this study was powered to detect a large change in the frequency of alarms, and was under-powered to detect subtle differences related to safety. Finally this study used one model of Auto-FiO_2_, application of these findings to other Auto-FiO_2_ systems ought to consider the construct of their alarm systems and their relative effectiveness at reducing prolonged events of extreme SpO_2_.

## Conclusion

The benefit of a looser approach in setting SpO_2_ alarm levels during Auto-FiO_2_ in this group of neonates is clear. Importantly it suggests the possibility of reducing the risk associated with alarm fatigue with the implementation of Auto-FiO_2_ with appropriate alarm levels. We conclude that the paradigms of alarm strategies used during manual titration of FiO_2_ need to be reconsidered when using Auto-FiO_2_ systems. We speculate that with reconsidered optimal settings, false positive SpO_2_ alarms can be minimized with better vigilance of clinically relevant alarms. Such changes in strategy should be prospectively evaluated as part of process improvement initiatives.

## Additional file

Additional file 1: CONSORT 2010 checklist of information to include when reporting a randomised trial*. (DOCX 155 kb)

## Abbreviations

Auto-FiO_2_: Automated control of inspired oxygen
FiO_2_: Fraction of inspired oxygen
NICU: Neonatal intensive care unit
SpO_2_: Arterial oxygen saturation measured noninvasively
